# Survival and Diversity of Human Homologous Dietary MicroRNAs in Conventionally Cooked Top Sirloin and Dried Bovine Tissue Extracts

**DOI:** 10.1371/journal.pone.0138275

**Published:** 2015-09-22

**Authors:** Joseph T. Dever, Michael Q. Kemp, Amber L. Thompson, Hana G. K. Keller, James C. Waksmonski, Chris D. Scholl, David M. Barnes

**Affiliations:** Research and Development Department, Standard Process, Inc., Palmyra, Wisconsin, United States of America; Kunming University of Science and Technology, CHINA

## Abstract

Dietary microRNAs (miRNAs), notably those found in milk, are currently being investigated for their potential to elicit biological effects via canonical binding to human messenger RNA targets once ingested. Besides milk, beef and other bovine tissue-derived ingredients could also be a relevant source of potentially bioactive dietary miRNAs. In this study, we characterized the human homologous miRNA profiles in food-grade, bovine-sourced sirloin, heart and adrenal tissue (raw, cooked, and pasteurized, freeze-dried extracts) via deep-sequencing and quantitative reverse transcription PCR (RT-qPCR). A total of 198 human homologous miRNAs were detected at 10 or more normalized reads in all replicates (n = 3) of at least one preparation method. Tissue origin rather than preparation method was the major differentiating factor of miRNA profiles, and adrenal-based miRNA profiles were the most distinct. The ten most prevalent miRNAs in each tissue represented 71–93% of the total normalized counts for all annotated miRNAs. In cooked sirloin, the most abundant miRNAs were miR-10b-5p, (48.8% of total annotated miRNA reads) along with the muscle-specific miR-1 (24.1%) and miR-206 (4.8%). In dried heart extracts, miR-1 (17.0%), miR-100-5p (16.1%) and miR-99a-5p (11.0%) gave the highest normalized read counts. In dried adrenal extracts, miR-10b-5p (71.2%) was the most prominent followed by miR-143-3p (7.1%) and 146b-5p (3.7%). Sequencing results for five detected and two undetected miRNAs were successfully validated by RT-qPCR. We conclude that edible, bovine tissues contain unique profiles of human homologous dietary miRNAs that survive heat-based preparation methods.

## Introduction

MicroRNAs (miRNA) are a ubiquitous class of small non-coding RNA in plants and animals that inhibit the protein translation of messenger RNA (mRNA) through antisense binding [1]. Currently, over 2,500 human miRNAs are listed in miRBase (version 21) [2], and their regulatory effect on cellular and physiological processes is widespread. Mature, single-stranded miRNAs, typically 22 nucleotides in length, are derived from longer hairpin precursors through cleavage by Drosha and Dicer [1]. They are then bound to Argonaute 2 as part of the RNA-silencing complex that facilitates binding of miRNAs to their mRNA targets.

In 2012, Zhang and colleagues published a detailed set of experiments providing evidence that orally consumed miR-168a in rice could be absorbed into systemic circulation, enter the liver, bind to and decrease translation of low-density lipoprotein receptor adapter protein 1 mRNA, and decrease LDL removal from plasma [3]. These results contradicted long-held assumptions that orally ingested RNA was nutritionally irrelevant and sparked a flurry of scientific discussion and activity on the topic [4–10]. To date, three follow-up studies [5–7] investigating the oral absorption of several plant-specific miRNAs and one animal-specific miRNA (miR-21) [5] have reported negligible miRNA absorption. One study [10] did report serum and tissue detection of miR-172, a prominent miRNA in *Brassica* species, following oral intake of RNA from *Brassica oleracea* in mice, but with apparent low bioavailability (less than 4.5%). While these reports highlight the potential bioactivity of plant-based dietary miRNAs, studies investigating animal-based miRNAs are perhaps more compelling. Raw human and cow’s milk are rich sources of dietary miRNAs [11] many of which reside within milk exosomes which may increase their stability and bioavailability [12,13]. Uptake of dietary miRNAs from cow’s milk into the systemic circulation of five healthy adults has now been demonstrated [14]. Milk-based miRNAs with immunomodulatory activity could even explain why consumption of raw cow’s milk in the first year of life is associated with a reduced incidence of atopic allergies [15]. Besides milk, little is known about the profile and potential bioactivity of miRNAs in other edible products of animal origin such as beef, which is consumed by millions of people worldwide [16], or bovine tissue extracts, which are used as dietary ingredients.

In this study, we utilized deep miRNA sequencing and quantitative reverse transcription PCR (RT-qPCR) to characterize the profile and stability of human homologous bovine miRNAs in food-grade bovine tissues including top sirloin, heart, and adrenal. We tested the effect of conventional cooking (pan-frying) or pasteurization followed by freeze-drying of liquid tissue extracts on the tissue miRNAs. Our overall goal was to provide prerequisite compositional information required for the broader effort of determining whether meat-based miRNAs are nutritionally relevant.

## Methods

### Sample Collection

Bovine muscle and organ tissues investigated were United States Department of Agriculture grade and suitable for human consumption. Meat products were not aged and were derived from an aggregate of breeds. Freshly-cut top sirloin samples (12–15 pounds each) were obtained from grocery stores in south-central Wisconsin (WI) including Walmart Supercenter (Monona, WI), Piggly Wiggly (Cambridge, WI), and Pick ‘n Save (Fort Atkinson, WI). Bovine hearts (Long Prairie Packing, Long Prairie, MN, Federal Establishment #253) and bovine adrenals (Cargill Wyalusing, Wyalusing, PA, Federal Establishment #9400) were purchased directly from United States Department of Agriculture-inspected facilities. Each organ sample consisted of pooled tissues (12–15 pounds each) from multiple animals.

### Sample Preparation

Sirloin, heart, and adrenal samples were initially ground using an STX Turbo Force meat grinder. To prepare conventionally cooked tissue samples, the ground tissues were pan-fried (no pink meat remaining) using an electric skillet set to 350°F (177°C). To prepare dried tissue extracts, the ground tissue was processed into a liquid tissue extract using a lab scale method based on a production scale, food-grade extraction process (Standard Process, Inc, Palmyra, WI). The liquid tissue extract was then flash pasteurized at 72°C for 15 seconds and subsequently freeze-dried. The freeze-dried extracts were ground into a powder using a mortar and pestle. All samples were then stored at -20°C.

### RNA Extraction

Total RNA was extracted from each raw, cooked, and dried tissue using the *mir*Vana™ miRNA Isolation Kit (Life Technologies, Carlsbad, CA) based on the manufacturer’s instructions. Briefly, 100 mg of the raw or pan-fried samples and 25 mg of the dried extracts were sonicated for 5 minutes in 600 μL Lysis/Binding^TM^ solution, followed by an acid-phenol extraction. Total RNA was collected in a spin column and eluted in 50–100 uL Elution solution. Quantity and purity were determined using a Nanodrop ND-1000. RNA integrity was assessed using a Bioanalyzer (Agilent, CA). Samples were shipped on dry ice to Arraystar, Inc. (Rockville, MD) for sequencing.

### Deep miRNA Sequencing

Total RNA from each sample was used to prepare the miRNA sequencing library which included the following steps: 1) 3'-adapter ligation with T4 RNA ligase 2 (truncated); 2) 5'-adapter ligation with T4 RNA ligase; 3) cDNA synthesis with RT primer; 4) PCR amplification; 5) extraction and purification of ~135–155 bp PCR amplified fragments (correspond to ~15–35 nt small RNAs) from the PAGE gel. After the completed libraries were quantified with an Agilent 2100 Bioanalyzer, the DNA fragments in the libraries were denatured with 0.1M NaOH to generate single-stranded DNA molecules, captured on Illumina flow cells, amplified *in situ* and finally sequenced for 36 cycles on Illumina HiSeq® 2000 according to the manufacturer’s instructions. After sequencing images were generated, image analysis and base calling were performed using Off-Line Basecaller software (OLB V1.8.0). Subsequently, 3’ adapter sequences were trimmed from clean reads (reads that passed Solexa CHASTITY quality filter) and the reads shorter than 15nt were discarded. The 3’-adapter-trimmed-reads (> = 15nt) were aligned to the latest known cow and human reference miRNA precursor set (Sanger miRBase 20) using Novoalign (v2.07.11). Reads (counts < 2) were discarded when calculating the miRNA expression. In order to characterize the isomiR variability, any sequence that matched the miRNA precursors in the mature miRNA region ±4nt (no more than one mismatch) were accepted as mature miRNA isomiRs, which were grouped according to the 5-prime (5p) or 3-prime (3p) arm of the precursor hairpin.

### Quantitative reverse transcription PCR (RT-qPCR)

RT-qPCR for selected miRNAs was carried out using the Taqman® assay (Life Technologies). Total RNA (10 ng) was combined with 100 mM dNTPs, 50 U/uL reverse transcriptase (RT), 20 U/uL RNase inhibitor, buffer, RNase-free water, and primers per manufacturer’s instructions. Reverse transcription reactions were run using a Mastercycler® Gradient (Eppendorf, Germany) with the following parameters: 30 minutes at 16°C, 30 minutes at 42°C, 5 minutes at 85°C, followed by a hold at 4°C. Samples that were not used immediately were stored at -80°C. The product was combined with Taqman® Universal PCR Master Mix II (no UNG), water, and the appropriate probe per manufacturer’s instructions. Samples, as well as no template controls, were run in triplicate. The Applied Biosystems 7300 Real-Time PCR software was set up as follows: 10 minutes at 95°C, then 40 cycles of 15 seconds at 95°C followed by 60 seconds at 60°C. Threshold values were set at 0.2 after verifying that 0.2 was above the background noise and fell within the exponential phase across all samples.

### Data Analysis

Sequencing (percentage annotated reads and 22 Nt-enrichment levels) and RT-qPCR data were compared using ANOVA with Tukey’s honest significant difference test as the post-hoc analysis (p<0.05). For miRNA filtering and differential analysis, the normalized read counts of the most abundant isoform for each annotated miRNA was uploaded into GeneSpring Mass Profiler Professional software, Version 13.0 (Agilent Technologies, Santa Clara, CA) as a generic (.csv) file. The log_2_ transformed read counts were compared using a Pearson’s correlation heat map. Univariate analysis was carried out using ANOVA with Benjamini-Hochberg as the multiple testing correction and Tukey’s honest significant difference test as the post-hoc analysis method (p<0.05). Differentially detected miRNAs were then visualized using principle component analysis with 4 components utilized and hierarchical clustering analysis with the Pearson’s Centered algorithm as the distance metric and Ward’s method as the linkage rule.

## Results

### Experimental Design

**Fig 1:**
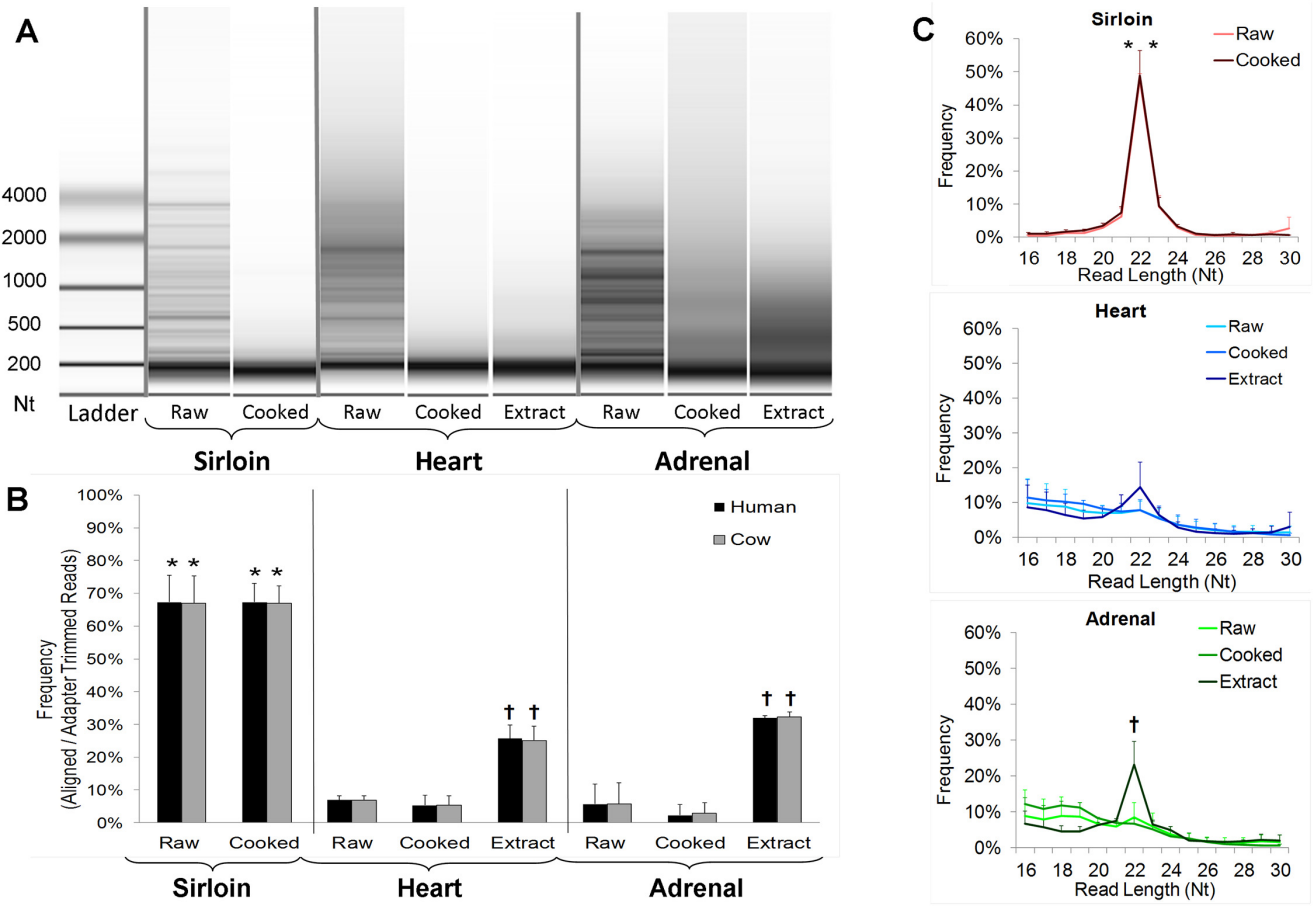
Sequencing Results. A) Representative images of total RNA from each raw and prepared tissue following electrophoretic separation. B) The percentage of adapter-trimmed reads annotated as bovine or human miRNAs. C) Read-length frequency graphs for raw and Prepared tissues. Data is expressed as mean ± SD, * significantly different (p<0.05) from all samples derived from different tissue types. † significantly different (p<0.05) from raw and cooked samples of the same tissue type.

Independent, commercial sources (n = 3) of top sirloin, heart, and adrenal were ground and prepared via pan-frying (cooking) or by a series of steps resulting in a pasteurized, freeze-dried extract (S1 Fig). Total RNA was then obtained from each sample for miRNA sequencing. We were unable to consistently obtain detectable RNA from dried sirloin extracts and so only raw and cooked sirloin RNA samples were sequenced. RNA integrity from all raw tissues was low and was further reduced in cooked samples and extracts (Fig 1A). Nevertheless, we proceeded with miRNA sequencing because previous studies have demonstrated that miRNAs are stable and measurable even in low-integrity RNA extracts subjected to high heat [17].

### Detection of human homologous bovine miRNAs

For each sample, 1 to 6.7 million clean reads corresponding to 0.5 to 5.8 million adapter-trimmed reads was obtained (S1 Table). In all cases, the number and percentage of adapter-trimmed reads aligned to known bovine miRNAs was nearly identical to the percentage aligned to known human miRNA sequences (Fig 1B). A much higher percentage of adapter-trimmed reads in raw (67 ± 8%) and cooked (67 ± 6%) sirloin corresponded to annotated cow and human miRNAs compared to heart and adrenal-based RNA samples. A higher miRNA annotation percentage was also observed in heart and adrenal-based dried extracts (26 ± 6% and 32% ± 11%, respectively) compared with their respective raw tissues (7 ± 1% and 6 ± 3%). Read length frequency analysis revealed similar trends. Raw and cooked sirloin were much more 22-Nt (nucleotide)-enriched (48 ± 2 and 49 ± 8%, respectively) compared with the other tissues (Fig 1C). An increased 22-Nt read length percentage was also noted in adrenal extracts (23 ± 6%) compared with raw adrenal (8 ± 4%).

### Dietary miRNA profiles are distinguished by tissue origin

**Fig 2:**
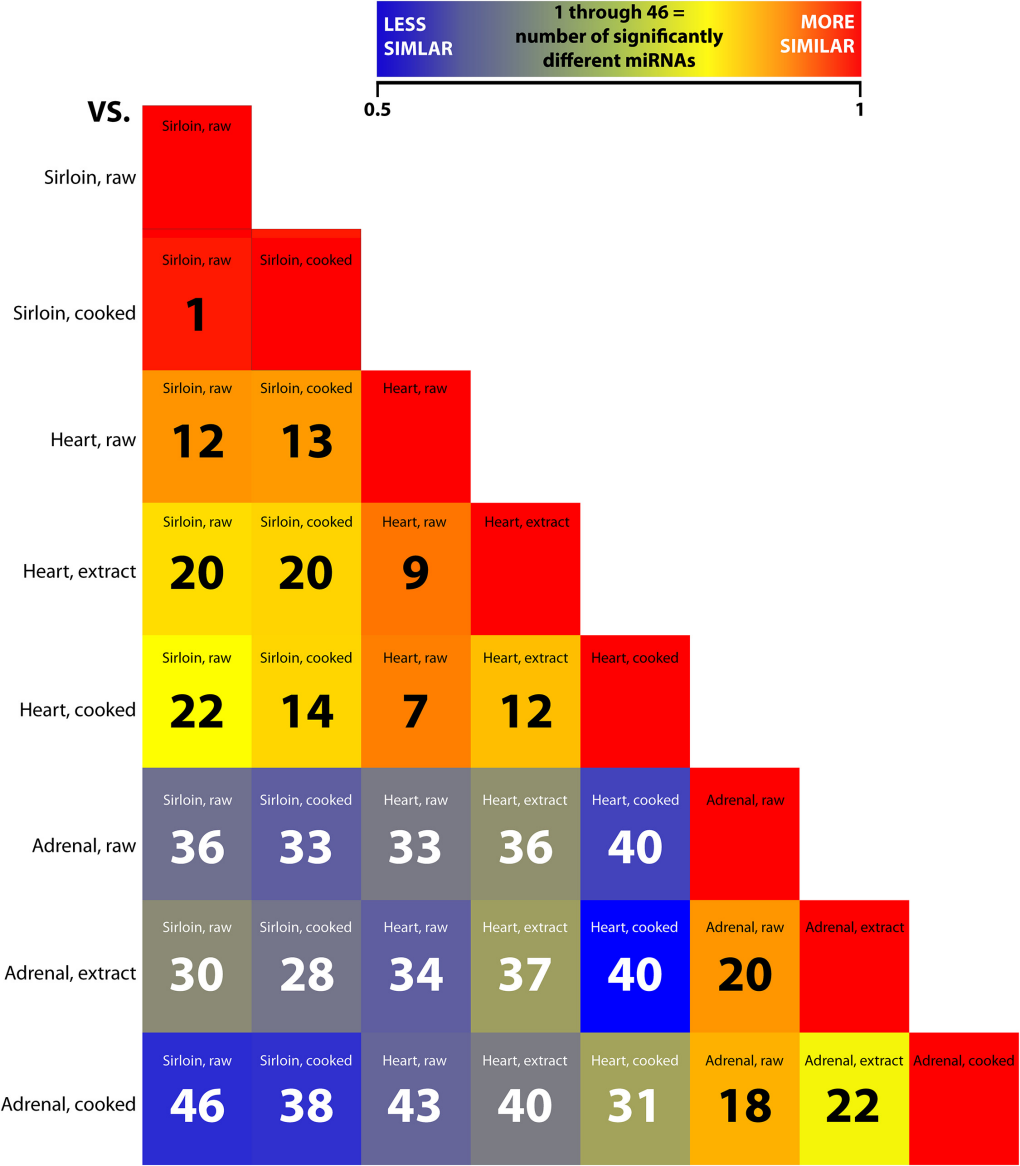
Correlation and Univariate Analysis of miRNA Profiles. Correlation heat map of the averaged log_2_ transformed normalized read counts of the 198 miRNAs detected at 10 or greater reads in all three replicates of at least one tissue and process. The number of significantly different miRNAs (p<0.05) between each comparison as determined by ANOVA is also indicated.

**Fig 3:**
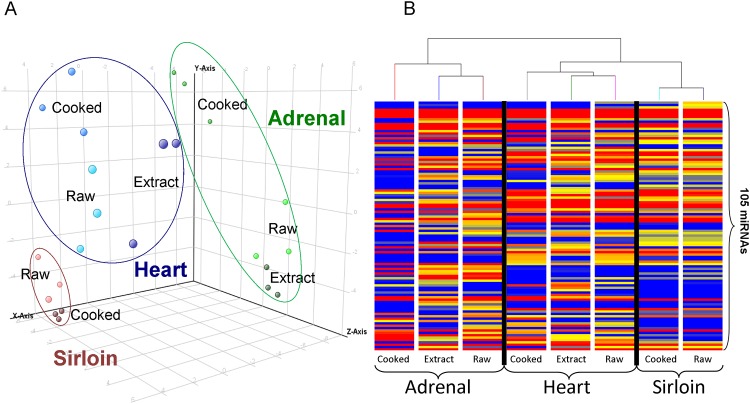
Multivariate Analysis of miRNA Profiles. A) Principle component analysis and B) hierarchical clustering of the log_2_ transformed normalized read counts of the 105 differentially detected miRNAs determined by ANOVA.

A total of 906 human-homologous miRNAs were initially detected (2 or greater normalized read counts of the most abundant miRNA isoform) across all samples. To select for the most consistent and robustly expressed miRNAs, those not detected at 10 or more normalized reads in all replicates (n = 3) of one or more sample groups were filtered. Normalized read counts of the resulting 198 miRNAs across all tissues and treatments were log_2_ transformed for differential analysis. Correlation among sample replicates for each group was highest for raw (0.92–0.95) and cooked (0.91–0.93) sirloin and ranged from 0.68–0.86 among replicates in heart and adrenal-based groups (S2 Table). Correlation and univariate statistical analysis (ANOVA) across experimental groups revealed that, regardless of processing, adrenal-based miRNA profiles were the most distinct showing lower correlation (0.47–0.63) and higher numbers of significantly different miRNAs (28–46) when compared with sirloin and heart-based miRNA profiles (Fig 2). In contrast, sirloin and heart-based miRNAs profiles, regardless of processing, were more correlated (0.72–0.84) and had lower numbers of significantly different miRNAs (12–22). In all, 105 miRNAs were found to be significantly different (p <0.05) in at least one post-hoc analysis across all comparisons. Principle component analysis of these 105 miRNAs revealed clear delineations in sirloin, heart, and adrenal-based miRNA profiles (Fig 3A). Differences between raw, cooked, and extract samples within each tissue were typically less distinct, but were still apparent. Hierarchical clustering of all averaged miRNA profiles effectively distinguished each tissue and process group (Fig 3B). The first main branch differentiated adrenal-based profiles from heart and sirloin-based profiles whereas heart and sirloin were separated on the second branch. In heart and adrenal, the miRNA profile of the raw tissue and extracts was clustered separately from the miRNA profile of the cooked tissues. Lower numbers of miRNAs present at 10 or greater reads in all replicates were detected within cooked heart (93) and cooked adrenal (61) compared to raw heart (114) and raw adrenal (113) whereas no similar loss was observed in heart extracts and adrenal extracts (122 and 114 miRNAs, respectively) (S2 Fig). Interestingly, there was no difference in the total number of consistently detected miRNAs in raw and cooked sirloin (111, and 115, respectively).

### Influence of tissue preparation methods on dietary miRNAs

**Fig 4:**
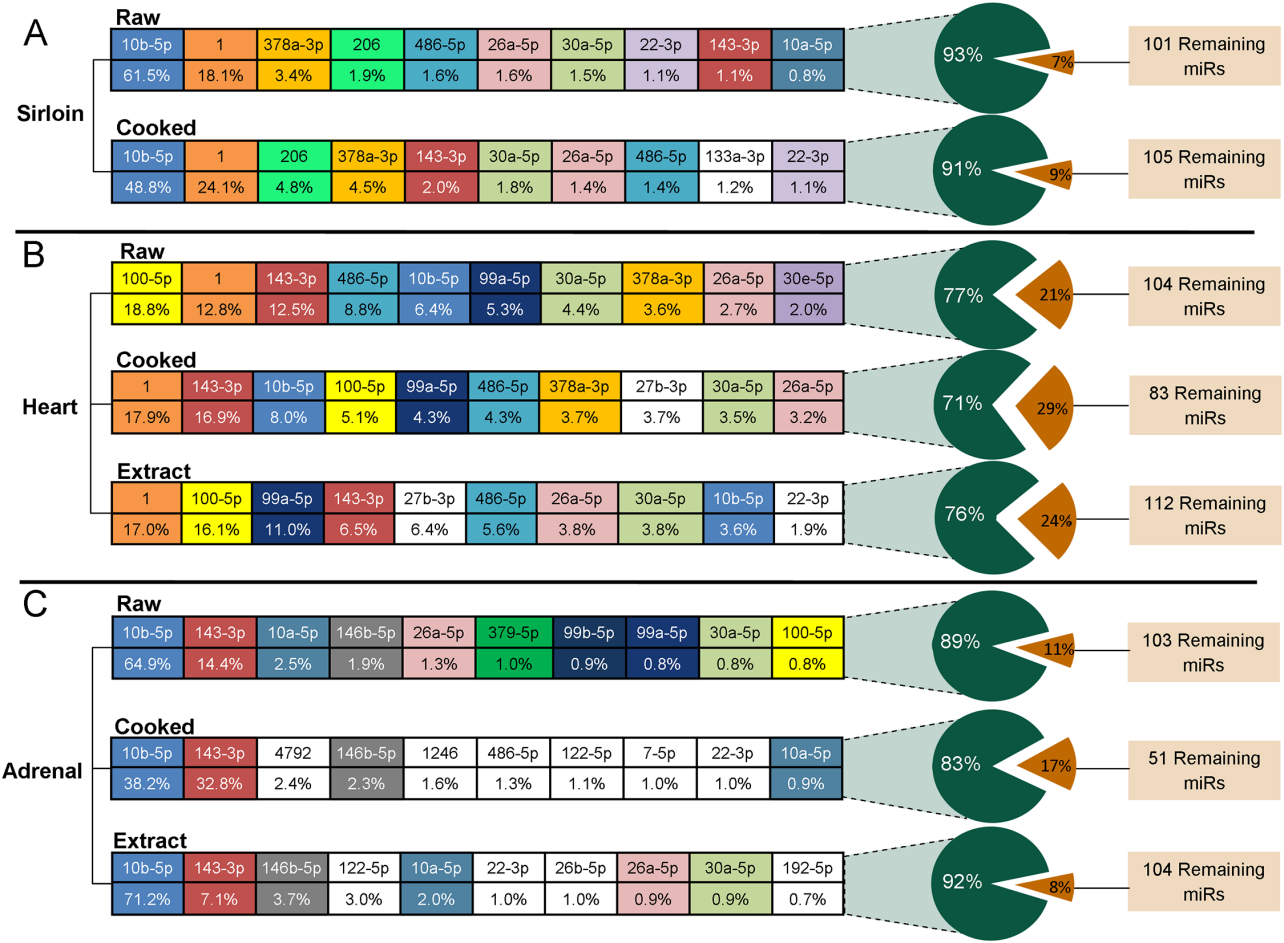
miRNA Profiles of Raw and Prepared Tissues. The average percentage contribution to the total human-annotated miRNA profile of the 10 most abundant miRNAs in each tissue and process group. Raw tissue miRNAs were each assigned a unique color. All miRNAs given a white background were present among the 10 most abundant miRNAs in cooked or dried extract samples but not the corresponding raw tissue.

We next examined the identities and relative contributions of specific human homologous miRNAs for each raw and prepared tissue. In all cases, the ten most abundant miRNAs accounted for the majority (71–93%) of the total miRNA-annotated averaged normalized reads (S3 Table, Fig 4). The miRNA profiles of cooked sirloin, cooked heart, and heart extract were very similar to their respective raw tissues. In contrast, the profile of the 10 most abundant miRNAs in cooked adrenal and adrenal extract differed somewhat from raw adrenal containing roughly half of the same 10 most abundant raw tissue miRNAs. Two miRNAs, miR-10b-5p and miR-143-3p, were among the most expressed miRNAs in all preparations of all three tissues while miR-26a-5p and miR-30a-5p were also in the top ten miRNAs in all groups except cooked adrenal. The muscle-specific miR-1 was prominent in sirloin and heart-based preparations while miR-206, another muscle-specific miRNA, was prevalent in both raw and cooked sirloin. In all adrenal-based groups, miR-146b-5p was detected at higher reader counts compared with sirloin and heart groups.

### Quantitative reverse transcription PCR (RT-qPCR) validation of deep sequencing results

**Fig 5:**
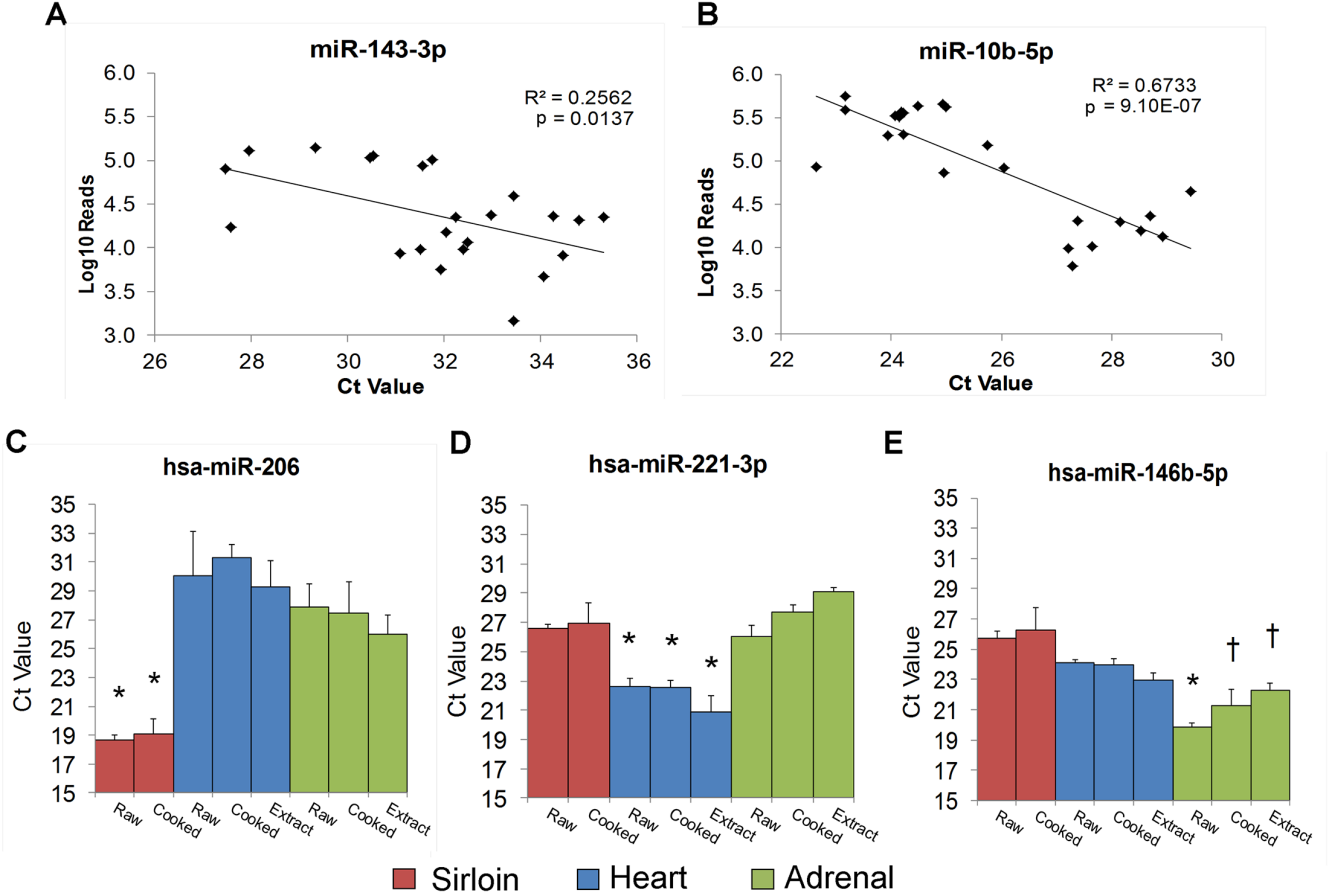
RT-qPCR Validation of Sequencing Results. A) Pearson’s correlation analysis of log_10_ normalized sequencing reads with C_t_ values for miR-10b-5p and miR-143-3p across tissue and process groups. B) C_t_ values for miR-206, miR-221-3p, and miR-146-5p across tissue and process groups. Data is expressed as mean ± SD, * significantly different (p<0.05) from all samples derived from different tissue types. † significantly different (p<0.05) from raw and cooked sirloin only.

Sequencing results from the raw and prepared tissues were validated by RT-qPCR using Taqman® assays. We first validated two miRNAs (miR-10b-5p, and miR-143-3p) that were ubiquitously detected in all sequenced samples. A significant correlation was observed between the log_10_-transformed read counts (3.2–5.7) and the Ct values (22.6–35.3) for both of these miRNAs (Fig 5). We also validated three miRNAs found at higher read counts in sirloin (miR-206), heart (miR-221-3p), and adrenal (miR-146b-5p). In each case, lower Ct values (corresponding to higher expression) were obtained in the expected tissue (Fig 5). Finally we validated two miRNAs (miR-506 and miR-889) that were not detected in any sample by sequencing. As expected, RT-qPCR analysis of these miRNAs in sirloin, heart, and adrenal-based raw and prepared samples confirmed the absence or near absence of these miRNAs (Ct values of greater than 36 or undetermined).

## Discussion

The publication of a surprising 2012 research article by Zhang and colleagues [3], which provided the first direct evidence for the absorption and bioactivity of a rice-based dietary miRNA in mice, awakened the broader scientific community to the paradigm-altering notion that miRNAs within food might have nutritional relevance. Several follow-up studies, also examining primarily rice and vegetable miRNAs, have had limited success in expanding on these findings [5–10]. In contrast, investigations examining the potential nutritional bioactivity of animal-based miRNAs in milk were already occurring prior to Zhang’s article and continue to yield promising results [11–15]. Consumption of raw cow’s milk in early life is strongly associated with decreased incidence of atopic syndrome (the predisposition to certain allergic hypersensitivity reactions) and could be one reason for the lower incidence of atopic challenges among individuals raised in a traditional farm setting [18–20]. Immunomodulatory miRNAs including miR-155, miR-146a, and miR-21 have now been proposed as bioactive molecules that elicit the atopy-protective effects of raw milk and colostrum by promoting the maturation of demethylated CD4^+^CD25^+^FoxP3^+^ regulatory T cells [15]. This hypothesis has now been further bolstered by recent findings that bovine miRNAs from milk are indeed absorbed by mice as well as humans into the systemic circulation [14].

Here, we have shown via deep sequencing and RT-qPCR that, in addition to milk, diverse and tissue-specific patterns of human-homologous miRNAs are also present in both conventionally cooked bovine meat and dried tissues. To our knowledge, these findings represent the first effort to catalog the complete profile of human homologous dietary miRNAs within consumable animal tissues.

Our experimental strategy was designed to not only identify dietary miRNAs in edible bovine-based consumable products but also to determine the effect of common preparation methods on the miRNAs present in the raw tissues. In our study, miRNAs were found to survive both pan-frying or pasteurization with subsequent freeze-drying, but we observed some differences between the two processes. First, we found that conventional pan-frying led to a 20–50% reduction in the number of miRNAs detected at 10 or greater reads in all three replicates of heart and adrenal, though interestingly, no such reduction was observed in cooked sirloin. In contrast, processing of these tissues to a dried extract largely preserved the raw tissue miRNA profile. We utilized a skillet temperature of 350°F (177°C) which was chosen to model common pan-frying methods for beef products [21]. In comparison, liquid tissue extracts were flash pasteurized at 72°C for 15 s prior to freeze-drying. Thus, miRNAs present near the burger-skillet interface were exposed to higher heat and may have been more rapidly degraded. Second, we observed an unexpected increase in the percentage of adapter-trimmed reads annotated as known miRNAs and a corresponding 22 Nt enrichment in sequenced RNA from laboratory-made heart and adrenal dried extracts compared with their respective raw tissues. The reason for this enrichment is presently unclear, but warrants further study. Overall, our results are consistent with recent observations that endogenous miRNAs within tissue and biofluid samples are stable even under conditions of high heat or acidity [12,17]. In contrast, exogenous free miRNAs are rapidly degraded when placed into plasma [22]. The stability of endogenous miRNAs may stem from their association with high-density lipoproteins, exosomes, and Argonaute 2 [12, 23,24]. Such interactions may have contributed to the observed stability of native miRNAs within tissues during heat treatment.

Two miRNAs, miR-10b-5p and miR-143-3p, were found at high levels in nearly all tested samples. Previous studies have found that miR-10b-5p targets Hox genes [25] while miR-143-3p helps to regulate cardiac morphogenesis [26]. Our findings of their ubiquitous expression in adult tissues suggest they have some fundamental biological role beyond development. Other dietary miRNAs that we detected at high levels do have known functions specific to the tissues in which they were observed. In sirloin and heart, several muscle-specific miRNAs (myomiRs) were observed: including miR-1, essential for the development and homeostasis of smooth and skeletal muscle [27,28], miR-378a-3p, involved in exercise-induced muscle hypertrophy [29], and miR-486-5p, which is also modulated by exercise and helps regulate the differentiation of myoblasts [30]. Sirloin contained the most abundant levels of miR-206, a well-studied and prominent skeletal muscle-specific miRNA [31], whereas heart tissue contained higher relative levels of miR-99a-5p and miR-100-5p. Both of these miRNAs target the Akt-mTOR signaling pathway, which regulates muscle protein synthesis [32]. Less is known regarding adrenal-specific miRNAs, however, higher relative levels of miR-146b-5b, which has been previously detected in adrenal tissue [33], were observed. The function of this miRNA within adrenal has not been studied.

Increasing evidence suggests that absorption of dietary miRNAs into the systemic circulation following oral intake may not always be efficient in mammals [5–7,10]. Those protected within exosomes or bound to proteins may be more stable and bioavailable [11–15, 22–24]. Even if they are not well absorbed into systemic circulation, dietary miRNAs could affect the gut itself. For example, immunologically-based dietary miRNAs, such as those found in milk, could exert effects within the gut-associated lymphoid tissue.

One important limitation of the present study is that we have provided only a relative rather than absolute quantification of meat-based miRNAs. Our primary objective was a broad characterization of the stability and diversity of dietary miRNAs within edible animal tissues. These data can now be utilized as a foundation from which to select dietary miRNAs of particular interest for quantitative analysis, an effort that is now much more feasible with the advent of droplet digital PCR technology. Accurately determining the net dose of dietary miRNAs within edible animal products as well as their bioavailability will be essential to fully assess their nutritional relevance.

In conclusion, we have identified numerous human homologous dietary miRNAs within cooked top sirloin and dried bovine tissue extracts. Each tissue was found to contain a unique profile of miRNAs, and these profiles remained largely intact following conventional pan-frying and flash pasteurization. These miRNAs can be considered unique constituents of edible animal tissues, but further experiments will be necessary to determine their exact nutritional effects.

## Supporting Information

S1 Fig Experimental Design. (TIF)

S2 Fig miRNA Diversity in Raw and Prepared Bovine Tissues. (TIF)

S1 Table Overview of Sequencing Results. (DOCX)

S2 Table Replicate Correlation Coefficients. (DOCX)

S3 Table Human Homologous Bovine miRNAs in Raw and Prepared Bovine Tissues. (XLSX)
