# Supplementary figures and images for: Survival and Diversity of Human Homologous Dietary MicroRNAs in Conventionally Cooked Top Sirloin and Dried Bovine Tissue Extracts
Source: PLoS One. 2015 Sep 22;10(9):e0138275. doi: 10.1371/journal.pone.0138275 (PMC4578893; doi:10.1371/journal.pone.0138275)

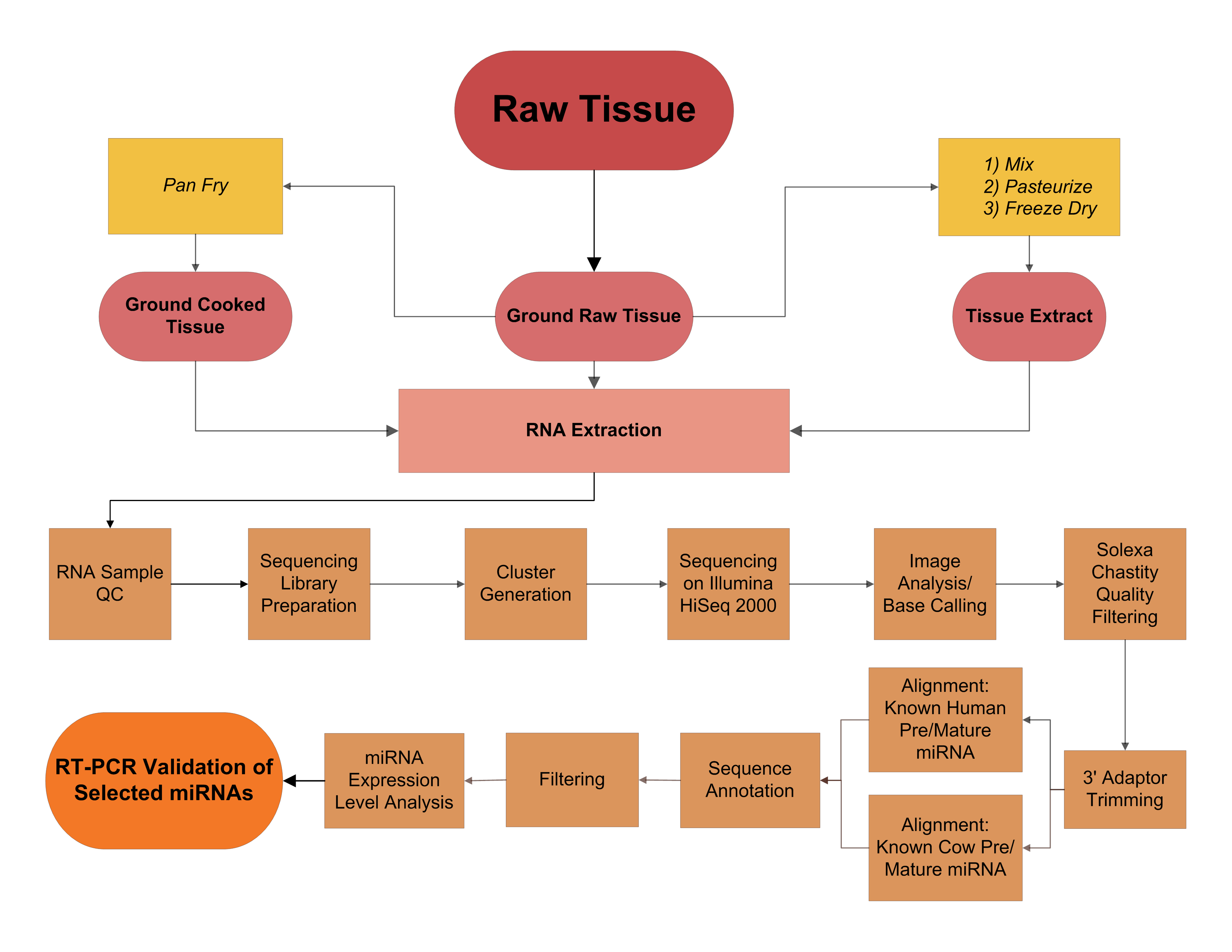

Supplement: S1 Fig — (TIF) [file pone.0138275.s001.tif]

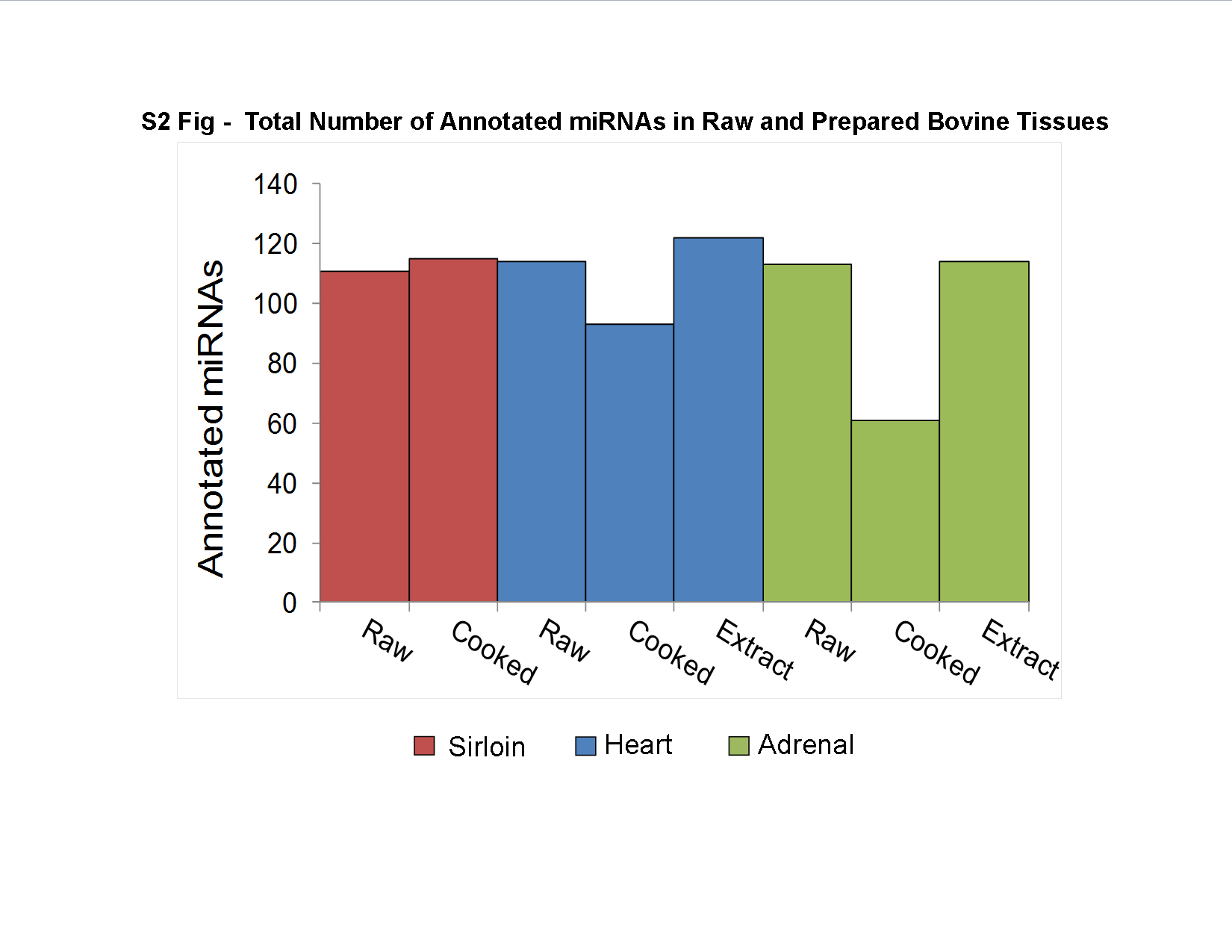

Supplement: S2 Fig — (TIF) [file pone.0138275.s002.tif]
