# Supplementary material for: Survival and Diversity of Human Homologous Dietary MicroRNAs in Conventionally Cooked Top Sirloin and Dried Bovine Tissue Extracts
Source: PLoS One. 2015 Sep 22;10(9):e0138275. doi: 10.1371/journal.pone.0138275 (PMC4578893; doi:10.1371/journal.pone.0138275)
Supplement: S1 Table — (DOCX) [file pone.0138275.s003.docx]

| **Sample Name** | **Clean Reads** | **Adapter-trimmed Reads (length >= 15nt)** | **Reads aligned to known cow pre-miRNA in miRBase 20** | **Reads aligned to known human pre-miRNA in miRBase 20** |
| --- | --- | --- | --- | --- |
| Sirloin Raw 1 | 2,430,300 | 2,336,196 | 1,372,724 | 1,385,246 |
| Sirloin Raw 2 | 5,349,483 | 5,265,496 | 3,539,310 | 3,531,067 |
| Sirloin Raw 3 | 3,945,708 | 3,864,855 | 2,907,499 | 2,925,117 |
| Sirloin Cooked 1 | 2,548,755 | 2,489,782 | 1,720,458 | 1,733,879 |
| Sirloin Cooked 2 | 5,850,533 | 5,755,090 | 3,506,167 | 3,498,358 |
| Sirloin Cooked 3 | 3,576,099 | 3,479,891 | 2,472,023 | 2,490,103 |
| Heart Raw 1 | 3,616,539 | 3,420,122 | 234,265 | 242,227 |
| Heart Raw 2 | 5,380,854 | 3,014,662 | 248,269 | 247,158 |
| Heart Raw 3 | 4,089,561 | 1,399,379 | 75,754 | 77,574 |
| Heart Cooked 1 | 3,287,798 | 992,894 | 32,139 | 34,305 |
| Heart Cooked 2 | 6,654,470 | 3,753,825 | 161,633 | 140,701 |
| Heart Cooked 3 | 5,525,113 | 3,897,938 | 337,040 | 342,475 |
| Heart Extract 1 | 3,297,488 | 2,895,330 | 782,354 | 795,821 |
| Heart Extract 2 | 4,638,349 | 1,614,570 | 490,872 | 497,594 |
| Heart Extract 3 | 3,374,388 | 487,359 | 87,527 | 91,465 |
| Adrenal Raw 1 | 2,583,145 | 1,519,956 | 110,617 | 110,962 |
| Adrenal Raw 2 | 6,039,673 | 2,123,548 | 40,919 | 38,354 |
| Adrenal Raw 3 | 4,322,256 | 4,078,777 | 322,762 | 319,547 |
| Adrenal Cooked 1 | 2,740,909 | 961,873 | 13,252 | 14,137 |
| Adrenal Cooked 2 | 1,009,467 | 400,452 | 17,021 | 9,585 |
| Adrenal Cooked 3 | 4,126,277 | 1,936,950 | 55,458 | 55,625 |
| Adrenal Extract 1 | 1,900,932 | 1,121,753 | 485,937 | 483,333 |
| Adrenal Extract 2 | 3,717,830 | 1,800,617 | 399,061 | 383,872 |
| Adrenal Extract 3 | 3,226,650 | 2,669,914 | 843,993 | 840,860 |
