# Supplementary material for: Survival and Diversity of Human Homologous Dietary MicroRNAs in Conventionally Cooked Top Sirloin and Dried Bovine Tissue Extracts
Source: PLoS One. 2015 Sep 22;10(9):e0138275. doi: 10.1371/journal.pone.0138275 (PMC4578893; doi:10.1371/journal.pone.0138275)
Supplement: S2 Table — (DOCX) [file pone.0138275.s004.docx]

S2 Table: Replicate Correlation Coefficients

| Replicate  Comparison | Sirloin | | Heart | | | Adrenal | | |
| --- | --- | --- | --- | --- | --- | --- | --- | --- |
|  | Raw | Cooked | Raw | Cooked | Extract | Raw | Cooked | Extract |
| 1 vs 2 | 0.92 | 0.91 | 0.87 | 0.75 | 0.85 | 0.69 | 0.68 | 0.80 |
| 1 vs 3 | 0.95 | 0.93 | 0.74 | 0.73 | 0.81 | 0.85 | 0.73 | 0.86 |
| 2 vs 3 | 0.94 | 0.92 | 0.76 | 0.80 | 0.83 | 0.68 | 0.68 | 0.85 |
